# Influence of age on the occurrence of adverse events in rheumatic patients at the onset of biological treatment: data from the BIOBADASER III register

**DOI:** 10.1186/s13075-020-02231-x

**Published:** 2020-06-15

**Authors:** Paloma Vela, Carlos Sanchez-Piedra, Carolina Perez-Garcia, María C. Castro-Villegas, Mercedes Freire, Lourdes Mateo, Cesar Díaz-Torné, Cristina Bohorquez, Juan M. Blanco-Madrigal, Inmaculada Ros-Vilamajo, Silvia Gómez, Rocio Caño, Fernando Sánchez-Alonso, Federico Díaz-González, Juan J. Gómez-Reino

**Affiliations:** 1 Rheumatology, Hospital General Universitario Alicante, Alicante, Spain; 2 ISABIAL, Alicante, Spain; 3 Unidad de Investigación SER, Madrid, Spain; 4 Rheumatology, Hospital del Mar, Barcelona, Spain; 5 Rheumatology, Hospital Reina Sofía, Córdoba, Spain; 6 Rheumatology, Hospital A Coruña, A Coruña, Spain; 7 Rheumatology, Hospital Germans Trias i Pujol, Barcelona, Spain; 8 Rheumatology, Hospital Santa Creu i Sant Pau, Barcelona, Spain; 9 Rheumatology, Hospital Príncipe de Asturias, Alcalá de Henares, Spain; 10 Rheumatology, Hospital de Basurto, Bilbao, Spain; 11 Rheumatology, Hospital Son Llatzer, Palma de Mallorca, Spain; 12 Departamento de Medicina Interna, Servicio de Reumatologia, Hospital Universitario de Canarias, Universidad de La Laguna, Calle Ofra s/n 38320, La Laguna, Santa Cruz de Tenerife, Spain; 13 Rheumatology, Hospital de Santiago, Santiago, Spain

**Keywords:** Rheumatoid arthritis, Ankylosing spondylitis, Psoriatic arthritis, Biologics, Elderly-onset rheumatoid arthritis

## Abstract

**Objectives:**

To assess whether age, at the beginning of biologic treatment, is associated with the time a first adverse event (AE) appears in patients with rheumatoid arthritis (RA), ankylosing spondylitis (AS), or psoriatic arthritis (PsA).

**Methods:**

All patients in the BIOBADASER registry diagnosed with RA, AS, and PsA, and classified as young (< 25 years old), adult (25–64 years old), elderly (65–75 years old) or very elderly (> 75 years old) at start of biological treatment were included. Factors associated with the appearance of a first AE using adjusted incidence rate ratios (IRR) (Poisson regression) were analyzed. Survival to first AE was studied by Kaplan-Meier analysis and hazard ratios (HR) by Cox regression.

**Results:**

2483 patients were included: 1126 RA, 680 PsA, and 677 AS. Age group stratification was as follows: 63 young, 2127 adults, 237 elderly, and 56 very elderly. Regression model revealed an increased probability of suffering a first AE at age 65 years or older [IRR elderly: 1.42 (CI95% 1.13–1.77)]. Other characteristics associated with AE were female gender, the use of DMARDs, including methotrexate, the presence of comorbidities, and the time of disease duration. Factors that had the greatest impact on survival over a first AE were age > 75 years [HR 1.50 (1.01–2.24)] and female gender [HR 1.42 (1.22–1.64)].

**Conclusion:**

Age at the start of treatment and female gender are key factors associated with the appearance of a first AE with biologics. Other factors related to patient status and treatment were also associated with a first AE in rheumatic patients treated with biologics.

## Introduction

Demographic trends are shaping the rapidly growing population aged 65 years and older throughout Western society [1]. This has enabled patients with chronic arthritis to reach advanced ages, as well as the appearance of rheumatic diseases in more elderly individuals. With regard to rheumatic diseases, an increasing number of patients are being diagnosed with elderly-onset rheumatoid arthritis (EORA) [2]. In 2017, a retrospective study [3] reported that the mean age of RA onset had significantly increased over the previous decade, from 55.8 years in 2002–2003 to 59.9 years in 2012–2013, with a corresponding shift in peak age from 50–59 to 60–69 years during that same time period. Although spondylarthropathies are generally encountered in young patients, all of the spondyloarthritis subgroups are represented in the elderly [4]. Although previous epidemiological series reported a prevalence for late-onset ankylosing spondylitis between 3 and 8% [5], the real prevalence of late-onset spondyloarthritis remains little known.

Regarding clinical manifestations, previous studies have found differences in EORA patients with respect to young-onset rheumatoid arthritis subjects in terms of the disease’s aggressiveness [6] and associated comorbidities, as well as poorer physical function, although the treatment provided the EORA groups was generally less intensive [7]. Most of these studies were carried out in Asiatic populations, with evidence on Caucasians being more limited. The late-onset group (> 50 years) of ankylosing spondylitis (AS) patients have exhibited greater neck and peripheral arthritis involvement than their younger counterparts [8]. .Moreover, in psoriatic arthritis (PsA) patients, elderly-onset has been linked to more severe manifestations and worse outcomes than in younger individuals [9, 10].

The risks that biological therapies pose in older patients have been previously evaluated. However, evidence remains scarce and mainly stems from clinical trials. In such studies, the elderly population is often underrepresented and usually only those with a few comorbidities are selected, achieving at best 12–22% in RA study groups [11], and only 5.3% and 1.4% in PsA and AS, respectively [12]. Gradually more data on biologic therapy in the elderly population are becoming available, but experience remains limited and is almost exclusively focused on tumor necrosis factor (TNF)-inhibitors(i) [13–16]. In this context, real-world evidence studies are needed to answer questions related to these older populations commonly excluded from clinical trials [17].

Our hypothesis is that patient age at the beginning of biological therapy is a risk factor for the early onset of a first adverse event (AE). Therefore, the aim of this work was to assess whether age at the beginning of the biological treatment (regardless of the initial biologic used) is associated with the time the first AE (regardless of its severity and outcome) appears in patients with RA, AS, or PsA. Other clinical factors, including gender and those related to concomitant medications, were also evaluated.

## Patients and methods

This was a multicenter prospective study with a real-world setting. Information was obtained from BIOBADASER, a national prospective registry of patients with rheumatic diseases treated with biologic disease-modifying antirheumatic drugs (bDMARDs), including biosimilars and targeted synthetic disease-modifying antirheumatic drugs (tsDMARDs), either with approved or off-label indications. BIOBADASER has been continuously collecting patient data since 2000. Its primary objective is assessing the safety of both bDMARDs and tsDMARDs. The previous version of BIOBADASER was updated in December 2015 and is known as BIOBADASER III [18]. With this update, an appraisal of the effectiveness of these treatments was added as a secondary objective. The registry protocol and materials of BIOBADASER III are available at http://biobadaser.ser.es. Briefly, all patients included in BIOBADASER are followed-up prospectively. The information of each patient is added to the registry at least once a year for treatment effectiveness issues, as well as every time that an AE or change in b/tsDMARDs treatment occurs. To assess both consistency and quality, the full database is monitored online annually; additionally, a sample of patient medical records are randomly selected and audited annually in situ by a specialized monitor at all 28 participating centers.

### Population

For this analysis, all patients included in BIOBADASER III with a diagnosis of either RA, PsA, or AS were selected and classified into four categories according to age at the start of biologics treatment: young (< 25 years old), adults (25–64 years old), elderly (65–75 years old), and very elderly (> 75 years old). Data extraction was conducted in December 2018. The analysis of this study includes data from 2000 to December 2018, and only those patients under active follow-up at the end of the study period were included. Signed informed consent was obtained from all patients included in the BIOBADASER III registry, covering all subsequent analysis including the present study.

This study was approved by the Ethics Committee (Hospital Clinic, Barcelona, Spain) and performed in accordance with Good Pharmacoepidemiology Practice Standards and with the principles of the Declaration of Helsinki.

### Outcome variables

The primary objective of this study was to evaluate the impact of age on the appearance of AEs at the beginning of biological treatments in patients with rheumatic diseases. AE was defined in BIOBADASER as any AE considered clinically relevant by the participating researcher. Those that required hospitalization or caused death were considered serious AEs. For the purpose of this analysis, we evaluated every AE independently of its severity. The following data were collected: (1) patient data, including gender, date of birth, diagnosis and date of diagnosis, comorbidities (Charlson index), and risk factors (smoking status); (2) data on treatment, duration of biologic treatment, types of biologics, and concomitant DMARD treatments; and (3) data on AE, including date of occurrence.

## Statistical analysis

Proportions, means, and standard deviations were used to describe the population. A Poisson regression model was carried out to explore the effects of, and other factors associated with, the appearance of a first AE age at the beginning of biological therapy. Crude and adjusted incidence rate ratios (IRRs) were calculated. Survival to first AE was studied by Cox regression models and Kaplan-Meier analysis. As both age and gender were associated with the risk of a first AE, we introduced an interaction term (age-sex) in the model. All analyses were performed using Stata version 13.1 (Stata Corp., College Station, TX 2013).

## Results

A total of 2483 patients were included in this study: 1126 (45.34%) RA, 680 (27.39%) PsA, and 677 (27.27%) AS. Baseline characteristics of patients by age group at onset of biological therapy are summarized in Table 1. A breakdown of the groups by age is as follows: 63 (2.53%) patients were young, 2127 (85.66%) adults, 237 (9.54%) elderly, and 56 (2.25%) very elderly. The percentage of women was greater in patients older than 65 years. Time of disease duration increased with age, while duration of biologic treatment decreased. Comorbidities assessed by the Charlson index increased with age, while smoking decreased (23.81% young, 30.32% adults, 27.00% elderly, and 12.50% very elderly). Methotrexate (MTX) was used similarly across all age groups (53.69% in the total sample), although treatment with corticosteroids increased with age (young 27.78%, adults 46.51%, elderly 64.18%, and very elderly 71.74%). When AE’s were analyzed, a tendency to increase their percentage with age was observed, both in the total and in the severe ones, but without reaching statistical significance.

**Table 1:** Characteristics of patients and first AE reported according to age group at onset of biological therapy

Variables	Young	Adult	Elderly	Very Elderly	***p***
***N*** **= 2483**	63	2127	237	56	
Age, mean (SD) in years	21.48 (4.48)	47.91 (9.98)	69.12 (2.87)	78.81 (3.10)	
Female gender (%)	25 (39.68)	1163 (54.68)	171 (72.159)	42 (75.09)	< 0.001
Disease duration, mean (SD) in years	2.72 (3.39)	7.28 (7.68)	9.46 (9.40)	8.19 (8.95)	< 0.001
Duration of biologic treatment, mean (SD) in years	3.12 (3.409)	2.60 (3.14)	1.79 (2.18)	1.57 (1.95)	< 0.001
**Diagnosis,*n*(%)**					
RA, *n* = 1126	13 (1.15)	903 (80.20)	161 (14.30)	49 (4.35)	< 0.001
PsA, *n* = 680	35 (5.15)	610 (89.71)	32 (4.71)	3 (0.44)	
AS, *n* = 677	15 (2.22)	614 (90.69)	44 (6.50)	4 (0.59)	
**Comorbidities**					
Charlson Index, mean (SD)	1.02 (0.13)	1.25 (0.68)	1.81 (1.49)	2.07 (1.41)	< 0.001
Tobacco: smokers/past smokers, *n* (%)	15 (23.81)	645 (30.32)	64 (27.00)	7 (12.50)	0.047
**Concomitant immunosuppressive treatment,*n*(%) of use**					
MTX	23 (41.8)	943 (53.61)	106 (55.79)	27 (60.00)	0.243
Corticosteroids	15 (27.78)	806 (46.51)	129 (64.18)	33 (71.74)	< 0.001
Initial corticoids dose (mg/day)	15.55 (16.26)	8.29 (7.24)	8.03 (5.85)	8.03 (6.37)	0.011
Other	8 (12.70)	627 (29.48)	83 (35.02)	23 (41.07)	0.002
**First AE (Total),*n*(%)**	33 (52.4)	1227 (57.7)	147 (62.0)	37 (66.1)	
Infections	9 (27.27)	391 (31.87)	50 (34.01)	11 (29.73)	0.789
Traumatic injuries	2 (6.06)	104 (8.48)	14 (9.52)	3 (8.11)	
Neoplasms	0 (0.00)	28 (2.28)	6 (4.08)	1 (2.70)	
Skin disorders	5 (15.15)	131 (10.68)	15 (10.20)	1 (2.70)	
Others	17 (51.52)	573 (46.70)	62 (42.18)	21 (56.76)	
**First severe AE,*n*(%)**	4 (6.3)	138 (6.5)	36 (15.2)	11 (19.6)	
Infections	1 (25.00)	45 (32.61)	13 (36.11)	1 (9.09)	0.052
Traumatic injuries	0 (0.00)	9 (6.52)	2 (5.56)	1 (9.09)	
Neoplasms	0 (0.00)	17 (12.32)	3.8 (8.33)	0 (0.00)	
Skin disorders	0 (0.00)	0 (0.00)	0 (0.00)	1 (9.09)	
Others	3 (75.00)	67 (48.55)	18 (50.00)	8 (72.73)	
**No AE*n*(%)**	30 (47.6)	900 (42.3)	90 (38.0)	19 (33.9)	

*AS* ankylosing spondylitis, *MTX* methotrexate, *PsA* psoriatic arthritis, *RA* rheumatoid arthritis, *TNF-i* tumor necrosis factor inhibitor

### Factors related to the incidence of the first AE

We initially analyzed all patients independently of the diagnosis. Table 2 shows the increased probability of suffering a first AE in relation to age at the beginning of biological treatment [IRR for elderly, 1.42 (CI95% 1.13–1.77), and IRR for the very elderly, 1.89 (CI95%1.27–2.81)], female gender [IRR 1.43 (CI95% 1.23–1.66)], the use of MTX [IRR: 1.40 (CI95% 1.22–1.61)], and IRR for other DMARDs, 1.29 [CI95% 1.12–1.50], but not in relation neither to the use of corticoids nor their initial dose in the adjusted regression models. Smoking habit was associated with a higher incidence of a first AE [IRR for current and past smokers, 1.31 (1.12–1.52)]. Comorbidities, as assessed by the Charlson Index, also significantly increased the likelihood of a first AE in the same adjusted model [IRR, 1.14 (CI95% 1.07–1.22)]. Supplementary Table 1 shows the interaction effect of age and gender. Data demonstrate that the effect of age on the appearance of AEs at the start of biological treatment occurs independently of gender.

**Table 2:** IRR of time to first AE. Poisson regression model crude and adjusted

Variables	Crude IRR (CI95%)	***p***	Adjusted IRR (CI95%)	***p***
**Age** (ref. adult)*				
Young	0.76 (0.54–1.09)	0.140	1.00 (0.66–1.53)	0.985
Elderly	1.61 (1.36–1.91)	< 0.001	1.42 (1.13–1.77)	0.002
Very elderly	2.01 (1.45–2.79)	< 0.001	1.89 (1.27–2.81)	0.002
**Female gender**	1.33 (1.20–1.49)	< 0.001	1.43 (1.23–1.66)	< 0.001
**Diagnosis (ref. RA)**				
AS	0.80 (0.70–0.91)	0.001	1.10 (0.91–1.33)	0.336
PsA	0.96 (0.84–1.09)	0.512	1.12 (0.94–1.33)	0.208
**Comorbidities**				
Charlson Index	1.15 (1.09–1.22)	< 0.001	1.14 (1.07–1.22)	< 0.001
Smoking (ref. No)				
Smoker/past smoker	1.25 (1.11–1.41)	< 0.001	1.31 (1.12–1.52)	0.001
**Treatments**				
Corticosteroids (ref. No)	1.31 (1.17–1.46)	< 0.001	1.1 (0.96–1.26)	0.192
Initial mean dose corticosteroids	1.00 (0.99–1.01)	0.842	0.99 (0.97–1.00)	0.100
MTX (ref. No)	1.41 (1.26–1.58)	< 0.001	1.40 (1.22–1.61)	< 0.001
Other DMARDs (ref. No)	1.17 (1.05–1.31)	0.006	1.29 (1.12–1.50)	0.001
**Time of disease duration**^&^	1.01 (1.00–1.01)	0.054	1.14 (1.07–1.22)	< 0.001

*AS* ankylosing spondylitis, *DMARDs* disease-modifying antirheumatic drugs, *IRR* incidence rate ratio, *MTX* methotrexate, *PsA* psoriatic arthritis, *RA* rheumatoid arthritis, *TNFi* tumor necrosis factor inhibitor

*Age at the beginning of biological treatment

Table 3 shows the same analyses stratified by diagnosis. Elderly patients exhibited an increased probability of suffering a first AE if they were diagnosed with RA and PsA, but not AS. In RA patients, comorbidities [IRR 1.08 (CI95% 1.00–1.17)] and a former smoking habit [IRR 1.28 (CI95% 1.05–1.57)] were associated with higher probabilities of suffering a first AE. In PsA patients, female gender [IRR 1.60 (CI95% 1.26–2.04)], as well as the use of MTX [IRR 1.31 (CI95% 1.01–1.68)], other DMARDs [IRR 1.43 (CI95% 1.09–1.88)], and corticosteroids [IRR 1.48 (CI95% 1.13–1.93)], was associated with a first AE. In AS, female gender [IRR 1.42 (CI95% 1.09–1.85)], the use of MTX [IRR 1.56 (CI95% 1.13–2.14)], the Charlson Index score [IRR 1.28 (CI95% 1.06–1.54)], and disease duration [IRR 1.02 (CI95% 1.01–1.03)] were associated with the appearance of a first AE.

**Table 3:** IRR of time to first AE stratified by diagnostic group. Poisson regression model, crude, and adjusted

Variable	RA				PsA				AS			
	Crude IRR (CI95%)	***p***	Adjusted IRR (CI95%)	***p***	Crude IRR (CI95%)	***p***	Adjusted IRR (CI95%)	***p***	Crude IRR (CI95%)	***p***	Adjusted IRR (CI95%)	***p***
Age (ref. adults)*												
Young	1.16 (0.62–2.17)	0.639	1.20 (0.62–2.33)	0.585	0.47 (0.19–1.13)	0.091	0.49 (0.18–1.31)	0.153	0.85 (0.52–1.39)	0.53	1.13 (0.67–1.90)	0.65
Elderly	1.59 (1.30–1.95)	< 0.001	1.55 (1.23–1.95)	< 0.001	1.60 (1.08–2.36)	0.019	1.25 (0.89–1.97)	0.336	1.36 (0.73–2.56)	0.336	1.34 (0.63–2.86)	0.446
Very Elderly	1.77 (1.24–2.52)	0.002	1.76 (1.18–2.61)	0.005	4.35 (1.40–13.57)	0.011	4.40 (1.30–14.87)	0.017	5.94 (1.48–23.84)	0.012	2.86 (0.65–12.59)	0.165
**Women**	1.00 (0.83–1.20)	0.99	1.14 (0.93–1.41)	0.209	1.72 (1.40–2.11)	< 0.001	1.60 (1.26–2.04)	< 0.001	1.35 (1.07–1.70)	0.012	1.42 (1.09–1.85)	0.01
**Treatments**												
Corticosteroids	1.01 (0.86–1.18)	0.913			1.73 (1.37–2.18)	< 0.001	1.48 (1.13–1.93)	0.004	1.59 (1.17–2.16)	0.003	1.35 (0.95–1.91)	0.098
MTX	1.26 (1.07–1.47)	0.004	1.25 (1.07–1.47)	0.006	1.34 (1.07–1.69)	0.012	1.31 (1.01–1.68)	0.041	1.78 (1.36–2.31)	< 0.001	1.56 (1.13–2.14)	0.006
Other	1.08 (0.93–1.25)	0.325			1.37 (1.09–1.72)	0.008	1.43 (1.09–1.88)	0.01	1.02 (0.76–1.36)	0.897		
**Smoking (ref. No)**												
Smoker/past smoker	1.29 (1.09–1.53)	0.004	1.28 (1.05–1.57)	0.015	1.36 (1.07–1.73)	0.012	1.09 (0.81–1.46)	0.578	1.26 (1.01–1.58)	0.042	1.18 (0.90–1.54)	0.234
**Comorbidities**												
Charlson Index	1.12 (1.05–1.20)	0.001	1.08 (1.00–1.17)	0.048	1.13 (1.00–1.29)	0.056	1.09 (0.93–1.29)	0.293	1.26 (1.07–1.47)	0.005	1.28 (1.06–1.54)	0.01
**Time**^**&**^	1.00 (0.99–1.01)	0.818			1.01 (0.99–1.03)	0.399			1.01 (1.00–1.03)	0.008	1.02 (1.01–1.03)	0.004

*Age at the beginning of biological treatment

^&^Time of disease duration

*AS* ankylosing spondylitis, *DMARDs* disease-modifying antirheumatic drugs, *IRR* incidence rate ratio, *MTX* methotrexate, *PsA* psoriatic arthritis, *RA* rheumatoid arthritis, *TNFi* tumor necrosis factor inhibitor

### Risk factors at the start of biological treatment on the time to a first AE

**Fig. 1:**
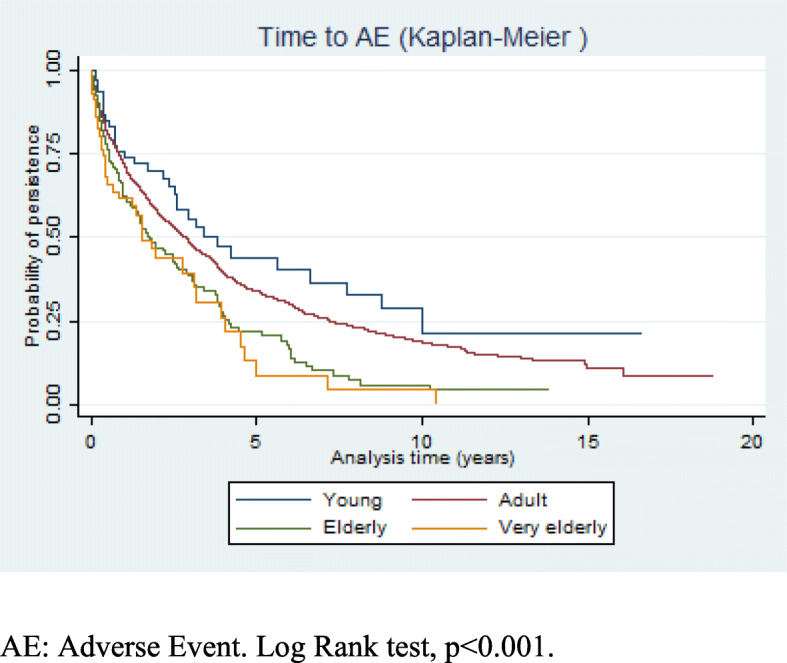
Survival analysis. Time to first AE. Kaplan-Meier graph. AE, adverse event. LogrRank test, *p* < 0.001

The time period until the appearance of a first AE was significantly longer in younger patients than in the adult, elderly, and very elderly groups (Fig. 1) (*p* < 0.001, log-rank test). Cox regression analysis (Table 4) shows that risk factors for the appearance of the first AE were age over 65 years (HR elderly 1.21, CI95% 0.97–1.52; HR very elderly 1.50, CI95% 1.01–2.24), female gender (HR 1.42, CI95% 1.22–1.64), the use of MTX (HR 1.21, CI95% 1.05–1.39), the presence of comorbidities (HR 1.13, CI95% 1.06–1.21), and disease duration (HR 1.01, CI95% 1.00–1.02). In our study, no significant differences were observed among the different rheumatic diseases studied in terms of the risk of suffering a first AE.

**Table 4:** Cox regression analysis

Variables	Hazard ratio (CI95%)	***p***
**Age (ref. adults)**		
Young	0.92 (0.60–1.40)	0.692
Elderly	1.21 (0.97–1.52)	0.089
Very Elderly	1.50 (1.01–2.24)	0.046
**Female gender**	1.42 (1.22–1.64)	< 0.001
**Diagnosis (ref. RA**)		
AS	0.93 (0.76–1.12)	0.432
PsA	0.96 (0.81–1.14)	0.668
**Comorbidities**		
Charlson index	1.13 (1.06–1.21)	< 0.001
Smoking (ref. No)		
Smoker/past smoker	1.16 (0.99–1.35)	0.059
**Treatments**		
Corticosteroids	1.04 (0.85–1.27)	0.699
Corticosteroid dose	0.99 (0.97–1.00)	0.140
MTX	1.21 (1.05–1.39)	0.008
Other DMARDs	1.10 (0.95–1.28)	0.182
**Disease duration**	1.01 (1.00–1.02)	0.007

*AS* ankylosing spondylitis, *CI95%* confidence interval 95%, *DMARDs* disease-modifying antirheumatic drugs, *HR* hazard ratios, *MTX* Methotrexate, *PsA* psoriatic arthritis, *RA* rheumatoid arthritis

## Discussion

The most important findings of this study can be summarized as follows: (1) age at the start of biologic treatment is the most important risk factor for the appearance of a first AE in RA, PsA, and EA patients and (2) female gender, the concomitant use of MTX, and the presence of comorbidities are also factors that increase said risk.

Our study consistently shows that the incidence of the first AE in rheumatic patients treated with biologics increases with age. Previous studies have shown the influence of age on the risk of AEs in patients treated with biologics. In addition, several studies are in agreement on the higher risk of serious infections (requiring hospitalization, intravenous antibiotics, or resulting in death) in older populations [12, 13, 19, 20], although this risk appears to be related to age itself.

Exactly how “elderly” is defined remains an important issue. Per convention, a person aged 65 years or more is often referred to as “elderly” [21]. However, elderly-onset rheumatoid arthritis (EORA) is defined as RA with an onset at age 60 years or over. Some groups have used the term “very old” to refer to people older than 75 or 80 years. It does make sense to consider separately those older than 75 years, taking into account that differences in both efficacy and safety responses have been found with older populations [22]. Thus, we decided to analyze independently people age 75 years or more (“very elderly”). Our results showed a progressive increase in the probability of having a first AE in relation to age at the onset of biological treatment. Variability between studies in age group definition complicates such comparisons, a problem that affects other fields in addition to rheumatology [23]. Our data also show that female gender is associated with higher risk of a first AE in patients treated with biologics, particularly in PsA. Some previous studies have found similar findings in drug retention rates in patients with PsA and AS [24]. However, the interaction model shows that gender does not seem to influence the effect of age at starting biologics on AEs.

Additionally, our study found that another factor contributing to the development of a first AE was the use of concomitant therapy, especially MTX. MTX’s effect was observed in patients generally and when stratified by diagnosis. Previous studies on elderly RA patients have reported findings consistent with our own [19, 20, 22, 23, 25], although some linked MTX toxicity mainly to renal function impairment rather than age itself [26]. As we do not have any renal function data, we cannot corroborate this observation. We found an increased use of corticosteroids with age, in agreement with previous studies [19, 27]. Although corticosteroid exposure appears to be an important predictor of infection [22, 28], in our study, neither the use of these compounds nor their initial dose did not seem to contribute to an initial AE. It is important to note that BIOBADASER does not record the dose of glucocorticoids administered at the time of the AE, information that could complement these findings. Overall, in terms of pathologies, no significant differences in the probability of a first AE were found, a finding in concordance with the previous report [29].

The current study has some limitations. First of all, most of our population (85%) were classified into the “adult” group, which reflects the prevalence typical of this disease type, predominantly one afflicting those in middle age [30]. Moreover, the “adult” group naturally corresponds to a higher age-range bracket. For all these reasons, we decided to classify patients according to their age at the start of biological therapy. Comparisons between studies in elderly patients are difficult due to a lack of consensus on defining age groups. Although EORA is usually defined as disease beginning at age 60 years or older, some authors classify as “elderly” those patients aged 65 years or older, differentiating as “very old” those older than 75 years [21, 31]. Second, we have evaluated those factors related to the appearance of a first AE, independently of the type of AE or their attendant consequences. Most published studies have analyzed the influence of age on treatment discontinuation or on the onset of serious AEs. Our study sought to study factors associated with the appearance of a first AE. However, one must bear in mind that the aging process is not experienced uniformly across the general population due to differences in genetics, lifestyle, and overall health [32]. In order to establish risk groups, the optimum classification parameter to use instead of chronological age would most likely be “frailty”, which is defined as a clinical syndrome in older adults, and one which carries an increased risk of poor health outcomes, including falls, disability incidents, hospitalizations, and increased mortality [33]. Although tools designed to measure frailty do exist, they were not included in BIOBADASER and therefore we could not incorporate them into the present study.

## Conclusions

Age at starting biological therapy is a key factor that seems to explain the appearance of a first AE in rheumatic patients. Other characteristics and clinical variables—such as female gender, the concomitant use of MTX, and the presence of comorbidities—are also factors that increase the risk of a first AE in this group of patients.

## Supplementary information

**Additional file 1: Table 1S.** Poisson regression model including the study of interaction sex-age. IRRs Adjusted.

## Data Availability

The data sets used and/or analyzed in the present study are available from the corresponding author on reasonable request.

## Abbreviations

AE: Adverse event
AS: Ankylosing spondylitis
bDMARDs: Biologic disease-modifying antirheumatic drugs
CI: Confidence interval
EORA: Elderly-onset rheumatoid arthritis
HR: Hazard ratios
IRR: Incidence rate ratios
MTX: Methotrexate
PsA: Psoriatic arthritis
RA: Rheumatoid arthritis
tsDMARDs: Targeted synthetic disease-modifying antirheumatic drugs
TNFi: Tumor necrosis factor inhibitors
