# Supplementary material for: Influence of age on the occurrence of adverse events in rheumatic patients at the onset of biological treatment: data from the BIOBADASER III register
Source: Arthritis Res Ther. 2020 Jun 15;22:143. doi: 10.1186/s13075-020-02231-x (PMC7296933; doi:10.1186/s13075-020-02231-x)
Supplement: Supplementary file 1 — Additional file 1: Table 1S. Poisson regression model including the study of interaction sex-age. IRRs Adjusted. [file 13075_2020_2231_MOESM1_ESM.docx]

Table 1S. Poisson regression model including the study of interaction sex-age. IRRs Adjusted.

| **Variable** | **IRR IC 95%** | **p** | |
| --- | --- | --- | --- |
| **Age (Ref. adults)** | | |  |
| Young | 1.05(0.61 - 1.80) | 0.865 | |
| Elderly | 1.54(1.02 - 2.32) | 0.038 | |
| Very elderly | 2.55(1.30 - 5.01) | 0.006 | |
| **Female gender** | 1.43(1.23 - 1.65) | <0.001 | |
| Young-Women | 0.99(0.46 - 2.10) | 0.973 | |
| Elderly-Women | 0.94(0.60 - 1.49) | 0.801 | |
| Very Elderly-Women | 0.67(0.30 - 1.50) | 0.333 | |
| **Diagnosis (ref. RA)** | | |  |
| AS | 1.05(0.87 - 1.27) | 0.611 | |
| PSA | 1.09(0.93 - 1.28) | 0.302 | |
| **Comorbidities** | | |  |
| Charlson-index | 1.10(1.02 - 1.18) | 0.014 | |
| Smoker/past smoker | 1.19(1.01 - 1.39) | 0.034 | |
| **Treatments** | | |  |
| Corticoids | 1.10(0.96 - 1.27) | 0.164 | |
| MTX | 1.35(1.18 - 1.54) | <0.001 | |
| Other DMARDs | 1.15(1.01 - 1.32) | 0.040 | |
| TNFi | 0.67(0.57 - 0.79) | <0.001 | |
| **Time of evolution of disease** | 1.01(1.00 - 1.02) | 0.023 | |

AS: Ankylosing Spondylitis; DMARDs: Disease-modifying anti-rheumatic drugs; IRR: Incidence rate ratio; MTX: Methotrexate; PsA: Psoriatic Arthritis; RA: Rheumatoid Arthritis; TNFi: tumour necrosis factor inhibitor.
